# xiSPEC: web-based visualization, analysis and sharing of proteomics data

**DOI:** 10.1093/nar/gky353

**Published:** 2018-05-08

**Authors:** Lars Kolbowski, Colin Combe, Juri Rappsilber

**Affiliations:** 1 Wellcome Centre for Cell Biology, School of Biological Sciences, University of Edinburgh, Edinburgh EH9 3BF, UK; 2 Bioanalytics, Institute of Biotechnology, Technische Universität Berlin, 13355 Berlin, Germany

## Abstract

We present xiSPEC, a standard compliant, next-generation web-based spectrum viewer for visualizing, analyzing and sharing mass spectrometry data. Peptide-spectrum matches from standard proteomics and cross-linking experiments are supported. xiSPEC is to date the only browser-based tool supporting the standardized file formats mzML and mzIdentML defined by the proteomics standards initiative. Users can either upload data directly or select files from the PRIDE data repository as input. xiSPEC allows users to save and share their datasets publicly or password protected for providing access to collaborators or readers and reviewers of manuscripts. The identification table features advanced interaction controls and spectra are presented in three interconnected views: (i) annotated mass spectrum, (ii) peptide sequence fragmentation key and (iii) quality control error plots of matched fragments. Highlighting or selecting data points in any view is represented in all other views. Views are interactive scalable vector graphic elements, which can be exported, e.g. for use in publication. xiSPEC allows for re-annotation of spectra for easy hypothesis testing by modifying input data. xiSPEC is freely accessible at http://spectrumviewer.org and the source code is openly available on https://github.com/Rappsilber-Laboratory/xiSPEC.

## INTRODUCTION

Mass spectra are the foundation of proteomics. Their analysis leads to identifications of peptides which in turn identify the proteins present in the sample (1,2). In the case of cross-linking experiments, linkage sites within the peptides are also identified (3,4). This provides proximity information for pairs of amino acid residues which can elucidate native protein structures (5) or protein–protein networks (6,7). In modern proteomics experiments thousands of spectra are generated, which necessitates automated algorithmic matching of spectra (search software). Nevertheless, humans still must be able to interact with spectra to remain in control of the identification process and investigate alternative hypotheses to those returned by automatic processing.

A typical proteomics dataset consists of two types of data: (i) mass spectra with associated data and (ii) peptides matched to the spectra by the search software. Both of these can come in different file formats depending on the manufacturer of the instrument or the developers of the search software, respectively. The multitude of file formats lead to an initiative for creating standardized formats for proteomics/mass spectrometry data by the Human Proteome Organization Proteomics Standards Initiative (HUPO-PSI). The HUPO-PSI standard format for encoding raw spectrometer output is mzML (8), with tools such as Proteowizard’s MSconvert (9) being available to convert virtually every mass spectrometry raw data format to mzML. The existing HUPO-PSI standard format for reporting identifications mzIdentML (10) has recently been updated to version 1.2.0 (11), adding support for cross-linking data. A variety of tools have been developed to convert legacy formats to mzIdentML (9,12,13).

We strongly encourage the shift toward the use of community wide consistent standard formats. Therefore xiSPEC is fully compliant with the newest PSI standard formats mzML and mzIdentML. To provide backward compatibility, we additionally support the still widely used Mascot Generic Format (MGF) (14) for peak list data and identifications in a comma-separated format. To the best of our knowledge, the only existing PSI compliant tool for viewing and analyzing spectra is PRIDE Inspector (15), which has the downside of requiring download prior to use. It does not currently support cross-link data, is not designed for hypothesis testing by modifying the peptide-spectrum match data and does not provide scalable vector graphic (SVG) output. Proteomics data including cross-links can be visualized and shared through the browser-based MS-Viewer (16). However, MS-Viewer lacks support for the PSI standard identifications format (mzIdentML) and the amenities of modern web development. Lorikeet (https://github.com/UWPR/Lorikeet) is used for spectrum visualization in proXL (17), a web-based platform for the analysis of cross-linking data. Lorikeet is equivalent in functionality to MS-Viewer, but with the benefit of being open source.

xiSPEC version 1.0 (website and sourceforge release 2012) is a stand alone browser-based spectrum viewer that allows interrogating single spectra and their interpretation in interconnected views with SVG output for figure making. It has replaced the original Mascot spectrum viewer since Mascot v. 2.5. We present here xiSPEC version 2.0, an interactive tool for visualizing and analyzing mass spectrometry data in the browser. It supports data from standard proteomics and cross-linking experiments. The interactive design of xiSPEC follows the principle of multiple coordinated views (18). The user has all information available on a single web page and all views of the data are interconnected. We hope that xiSPEC’s ease of use will positively impact on the proteomics community by enhancing data interrogation and sharing.

## IMPLEMENTATION

Essential to all the previously mentioned spectrum visualization tools is the ability to associate fragments of peptide sequences with peaks in the spectra. There are three ways the annotation of peaks with corresponding peptide fragments could occur. First, the annotated fragments could be recorded in the mzIdentML file, the specification allows for this. This has the benefit of allowing the spectrum viewer to show exactly those annotations that the search software used to derive the identification. Nevertheless, most search software do not include this information as it causes datasets to grow substantially. MS-Viewer and Lorikeet use an alternative approach by incorporating the annotation process into the spectrum viewer. At last, spectrum visualization and annotation can be separated into separate software components or services. This provides uniform annotation, separates concerns, eases the maintenance of both the annotation and visualization tools and allows the use of both independent of each other. An example of a stand-alone annotator is PRIDE-asap (19). PRIDE-asap does not support cross-linked peptides. Therefore, we use xiAnnnotator (https://github.com/Rappsilber-Laboratory/xiAnnotator).

xiSPEC itself consists of two major components: the data handling back-end and the interactive data visualization front-end. The backend data parser is written in python using the pymzML (20) (for mzML input) and pyteomics (21) (for mzIdentML input) packages and is also available as an open source project through GitHub (https://github.com/Rappsilber-Laboratory/xiSPEC_ms_parser). For fast data access into MGF files, xiSPEC uses an indexed based file reader we derived from pymzML. The parsed data gets written into a SQLite database. SQLite provides the benefit of having a single separate file for each dataset. This enables easy storage, deletion or compression of data. It is also cross-platform stable. On an annotation request, the data are read out from the database and converted into JSON. The annotation of spectra is done on-demand via API communication in JSON from the front-end to the Java application xiAnnotator running on a separate server. The front-end is written in JavaScript. The spectra data-visualization is based on D3 (22) to create SVGs. Event handling and synchronization between different views is achieved using the jQuery and Backbone JavaScript libraries. The interactive results tables are generated employing the DataTables jQuery library with server-side PHP processing for ordering, filtering and searching. This prevents processing of large datasets leading to prolonged load times and browser crashing.

## FUNCTIONALITY

### Data input

**Figure 1.**
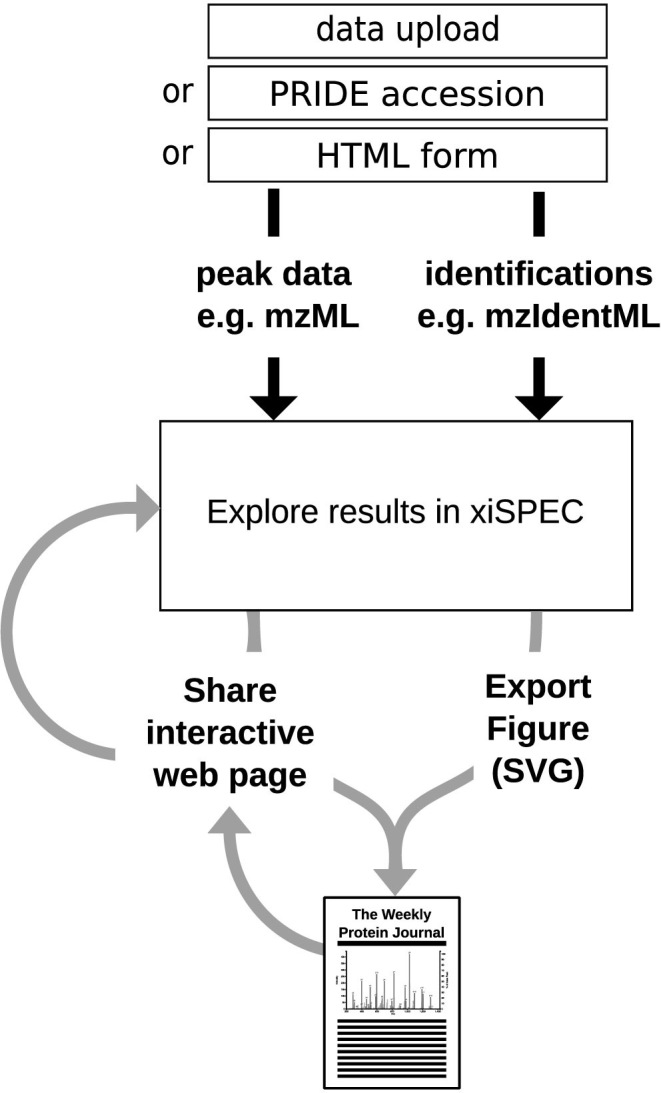
Overview of xiSPEC workflow. The input for xiSPEC are peak list data and peptide identifications. The user can either upload files directly to the xiSPEC server or select them from the PRIDE repository by providing the PXD accession number. For single spectra analysis data can be provided via HTML form input. Users can save datasets (publicly or password protected) and share them using a unique URL. Results can be exported as SVG for use in publications and presentations.

Users can provide data either by direct data upload of an identifications and peak list file(s) pair or by providing a PXD accession number to the PRIDE repository (23) and subsequent selection of the files from the list of project files (Figure 1). In the latter case, xiSPEC uses the PRIDE RESTful API (24) to retrieve the project files. After user selection the files are directly downloaded from the PRIDE FTP server to the xiSPEC backend server where they are processed. This relieves the user from downloading and then re-uploading the files, thus making data deposited in PRIDE accessible easier and independent of end-user internet connection speed. xiSPEC supports the PSI standard proteomics identifications file format mzidentML and additionally a simple csv file format for non-standard data. This format is described at http://spectrumviewer.org/help.php#csv (Supplementary Table S1). Supported peak list data formats are the PSI standard mzML and also MGF. xiSPEC supports compression archives in .gz and .zip formats. For single spectra analysis users can input data directly via an HTML form with interactive peptide preview. All three options are available through the Upload page of the website.

### Features

**Figure 2.**
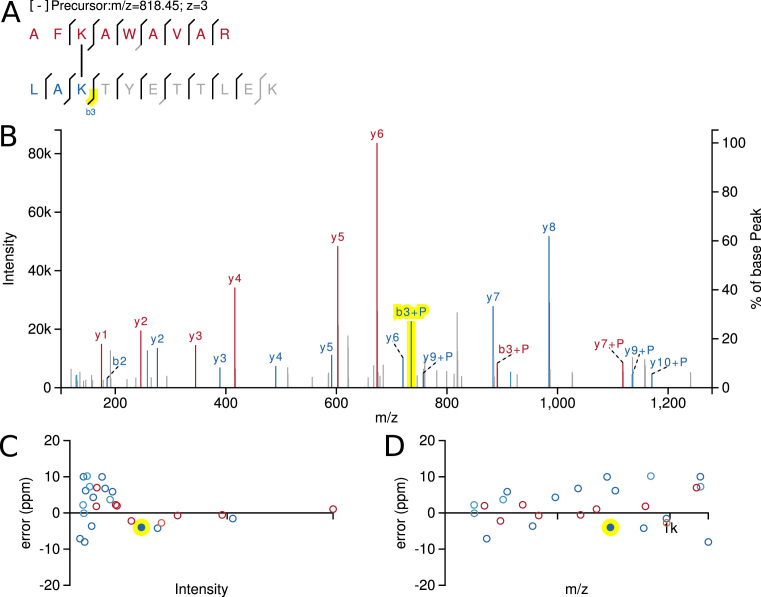
SVG output of xiSPEC’s views for a cross-linked peptide example. (**A**) Peptide sequence with fragmentation key. Amino acid residues in one-letter code. Lines show matched peptide fragments. Grayed-out residues are not included in currently selected fragment. (**B**) Annotated mass spectrum. Matched peaks are colored and labeled (though labels of neutral-loss fragments are hidden in this example). (**C**) and (**D**) show spectra QC plots. Each point represents a matched peak of the mass spectrum. (C) Fragment match error over peak intensity. (D) Match error over *m/z*. Coloring (red and blue) is used to differentiate between the two peptides. Neutral-loss fragments are displayed in a lighter color. Yellow highlight is the currently selected fragment (interconnected between all views).

Opening a dataset in xiSPEC presents the user all available information on a single web page with inter-connected views (Figure 2). Sub elements of the website layout can be re-sized or hidden when they are not needed to accommodate different use cases and individual users’ preferences.

Datasets can be saved for later access or for sharing with collaborators by using a unique URL. To save a dataset the user has to input a name for his dataset and chose whether it will be publicly available or private. If the user chooses private the user needs to choose a password that will be required to access his dataset. In this way the dataset can still be shared with collaborators or reviewers before making it publicly available.

Identification results are presented to the user in an interactive data table. Columns can be hidden to declutter the view of unnecessary information. Results are paginated to provide the user with the ability to view the results and plots at the same time. The order of results can be changed simply by clicking on the column name. If more than one score is present the user can select the score used for ordering via a drop down menu. Results can be filtered by string search. Additionally predefined filters are provided to toggle displaying decoy identifications, identifications not passing the threshold defined by the search software and to hide linear identifications (useful for cross-link datasets). If the identifications input file contains alternative explanations they can be easily accessed by switching to the ‘alternative explanations’ tab. This allows for quick comparison and manual reviewing of potential misidentifications. The protein column is automatically converted to a link to the UniProt (25) sub-page for the corresponding protein if the accession number is present in the input data.

The underlying data of the selected identification is presented to the user in multiple interactively connected views. The two main ones being the annotated mass spectrum (Figure 2A) with a peptide sequence fragmentation key (Figure 2B). Matched fragment peaks (and their isotope cluster peaks) are visualized through color and fragment name labels. For cross-linking data, two different colors are used to differentiate the two peptides. Neutral-loss fragments are displayed in a lighter color. Additionally, spectra quality control (QC) plots are generated to allow for manual identification quality assessment. They show the error of matched fragments plotted over intensity (Figure 2C) and over acquired *m/z* range (Figure 2D). All of these views are interactive SVG elements. Hovering over a data point in any view displays a tooltip with detailed information. Highlighting or selecting data points in any view is represented in all other views so that the information available in the different views can be leveraged together.

**Figure 3.**
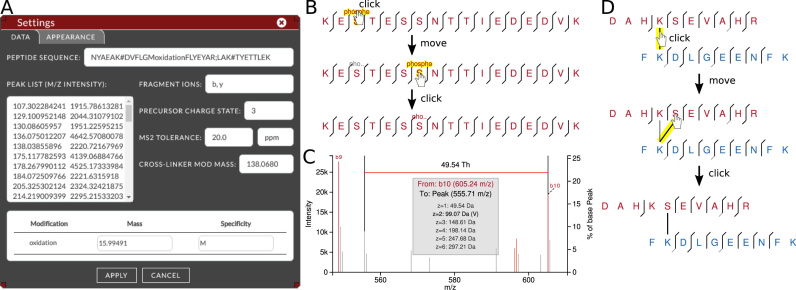
xiSPEC feature examples. (**A**) Settings view. Peptide input data can be modified in the displayed tab. Appearance customization can be done through the ‘appearance’ tab. (**B**) Changing PSM modification positions. (**C**) Use of measuring tool in zoomed-in excerpt of spectrum. Measure distance between peaks with automatic calculation and amino-acid residue matching for multiple charge states. (**D**) Changing cross-linker position.

Detailed instructions to xiSPEC’s features can be found on the help pages (http://spectrumviewer.org/help.php#features). They are described as text instructions while at the same time being displayed as GIFs to visually guide the user. xiSPEC includes the following features:

- Zooming into spectra and moving around the currently displayed section. The current zoom level (*m/z* range) can be locked, i.e. temporarily disabling the zoom and move functionality in the current spectrum. The selected *m/z* range stays in place when switching spectra to enable easy cross-spectra comparison of specific *m/z* regions.
- Changing appearance styles of the output by changing color schemes and highlight color (Figure 3A).
- Toggle display of neutral loss fragment labels, to declutter the annotated spectrum.
- Changing to absolute error values for the QC plots.
- Measuring distances between peaks in Thompson. The distance is converted to masses calculated for multiple charge states and possible amino acid matches are displayed (Figure 3C). This can be help analyzing unexplained peaks in the spectra.
- Option to move labels for better visibility. This click and drag functionality automatically creates dashed lines to the corresponding peak which simplifies figure making (Figure 3C).
- Adding new post-translational modifications (PSMs) or changing modification masses. This can be done through the data settings view, by typing non-uppercase characters into the peptide sequence input (Figure 3A). Modification masses can be changed in the modification table. Inserting a modification that is not present in the input data will result in adding a row to the table.
- Changing modification position. Modification positions can be moved from one residue to another by first clicking on the modification in the peptide sequence fragmentation key view and then clicking on the destination residue (Figure 3B). Alternatively, the modification can be moved in the input sequence (Figure 3A).
- Changing cross-linker positions can be done by simply clicking on the cross-linker line in the peptide sequence fragmentation key view and selecting the new destination residue (Figure 3D). Another way is to move the cross-link symbol (#) in the input sequence (Figure 3A). Specifications on the data input syntax can be found in the help pages.
- Modifying precursor data, namely the precursor charge state and the peptide sequence (amino acids and modifications) (Figure 3A).
- Changing fragment ion types considered. Ion types currently supported are unfragmented precursor (peptide) ion, b, c, y, z ions (Figure 3A).
- Changing the permitted error tolerance for matching fragment peaks (Figure 3A).
- Reverting back to original annotation after modifying input data at the click of a button (background color changes to visualize changed data).

### Data output

xiSPEC offers the user the option to download single plots in publication quality as easily modifiable vector graphics (Figure 2). Additionally, xiSPEC allows sharing of visualized datasets at the ease of just sharing an unique URL, open or password protected. Anyone using a modern web browser can access it without the need to install third party software.

## USE CASES

We imagine xiSPEC bringing a positive impact to individuals from a variety of different backgrounds working with mass spectrometry proteomics data. Users can be divided into two main groups: (i) providers of data and (ii) users of data.

Providers of data are for example staff of mass spectrometry core facilities. They are not necessarily interested in interpretation details, but have the obligation of communication, i.e. sharing the data with their users. Authors of publications that include mass spectrometry data also need a way to make their annotated data available to their reviewers and readers. In fact many proteomics field guidelines include making annotated mass spectra of published results available (26–28), which can be achieved using xiSPEC by either sharing the datasets unique URL or downloading plots for use in publication. When still looking at data scientists may benefit from the possibility of interactive sharing with collaborators, other scientists or the community. Another example are teachers and lecturers who want to give their students access to view and work with mass spectrometry data. Removing the extra step of having to download additional software lowers the entry barrier significantly.

Users of data, e.g. biologists who receive mass spectrometry data from core facilities often have no dedicated software installed. Installing and getting offline software to work constitutes an additional hurdle, often involving frustration from steep learning curves. We believe that xiSPEC with its ease of use, user-centered and browser-based approach can simplify both lives of biologists and core facility members. By providing intuitive and easy to use tools for testing of hypothesis (measuring tool, re-annotation with modified parameters and QC plots) we also see a benefit for mass spectrometrists diving deeper into their data when looking at non-standard results.

## CONCLUSION

The importance of data sharing is widely appreciated in proteomics and secured by initiatives like ProteomeXchange. However, accessing, sharing and analyzing individual spectra from proteomic datasets is very cumbersome. xiSPEC aims to fill this gap by placing the user at the center of the interface design offering multiple view synchronization and a multitude of other features for hypothesis testing and sharing. As an actively developed open-source tool, it is open to community feature requests and contribution. We will be working toward seamless integration with online repositories such as PRIDE, UniProt or INTACT for users to interrogate primary data through simple web browsing.

## DATA AVAILABILITY

xiSPEC is an open source collaborative initiative available in the GitHub repositories (front-end: https://github.com/Rappsilber-Laboratory/xiSPEC; back-end: https://github.com/Rappsilber-Laboratory/xiSPEC_ms_parser). xiAnnotator is an open source collaborative initiative available in the GitHub repository (https://github.com/Rappsilber-Laboratory/xiAnnotator). All of which are freely available under the Apache License v2.0.

## Supplementary Material

Supplementary Data
